# A quantitative MRI index for assessing the severity of hippocampal sclerosis in temporal lobe epilepsy

**DOI:** 10.1186/s12880-020-00440-z

**Published:** 2020-04-25

**Authors:** Wanchen Dou, Lei Zhao, Changbao Su, Qiang Lu, Qi Liu, Jinzhu Guo, Yuming Zhao, Yishan Luo, Lin Shi, Yiwei Zhang, Renzhi Wang, Feng Feng

**Affiliations:** 1 Department of Neurosurgery, Peking Union Medical College Hospital, Beijing, China; 2 BrainNow Research Institute, Shenzhen, Guangdong Province China; 3 Department of Neurology, Peking Union Medical College Hospital, Beijing, China; 4 Department of Imaging and Interventional Radiology, The Chinese University of Hong Kong, Hong Kong, SAR China; 5 Department of Radiology, Peking Union Medical College Hospital, Beijing, China

**Keywords:** Temporal lobe epilepsy, Hippocampal sclerosis, Grading scale, Quantitative MRI, HSI

## Abstract

**Background:**

Hippocampal sclerosis (HS) is associated with post-surgery outcome in patients with temporal lobe epilepsy (TLE), and an automated method that quantifies HS severity is still lacking. Here, we aim to propose an MRI-based HS index (HSI) that integrates hippocampal volume and FLAIR signal to measure the severity of HS.

**Methods:**

Forty-two pre-surgery TLE patients were included retrospectively, with T1-weighted (T1W) and FLAIR images acquired from each subject. Two experienced neurosurgeons (W.D. and C.S.) and one neurologist (Q.L.) rated HS severity with a four-class grading scale (normal, mild, moderate and severe) based on both hippocampal volume loss and increased FLAIR signal. A consensus of HS severity for each subject was made by voting among the three visual rating results. Regarding the automatic quantification, the hippocampal volume was quantified by AccuBrain on T1W image, and the FLAIR signal of hippocampus was calculated as the mean intensity of hippocampal region on the FLAIR image (normalized by the mean intensity of gray matter). To fit the HSI from visual rating, we applied ordinal regression with the voted visual rating as the dependent variable, and hippocampal volume and FLAIR signal as the independent variables. The HSI was calculated by weighting the predicted probabilities of the four-class grading scales from ordinal regression.

**Results:**

The intra-class correlation coefficient (single measure) of the three raters was 0.806. The generated HSI was significantly correlated with the visual rating scales of the three raters (W.D.: 0.823, Q.L.: 0.817, C.S.: 0.717). HSI scores well differentiated the different HS categories as defined by the agreed HS visual rating (normal vs. mild: *p* < 0.001, mild vs. moderate: *p* < 0.001, moderate vs. severe: *p* = 0.001).

**Conclusions:**

The proposed HSI was consistent with visual rating scales from epileptologists and sensitive to HS severity. This MRI-based index may help to evaluate HS severity in clinical practice. Further validations are needed to associate HSI with post-surgery outcomes.

## Background

Hippocampal sclerosis (HS) is the most common pathology underlying medically intractable temporal lobe epilepsy (TLE) [1]. The best option to enable the TLE patients seizure-free is surgical resection, especially when HS is present [2]. Moreover, HS severity was also found to associate with the long-term post-surgery outcome of TLE [3]. In general, HS is confirmed with histopathological specimens from the resected tissue, where an international consensus of HS classification (ILAE) has also been built in the recent years [4]. However, the neuropathological examinations are invasive, and the presence of HS can only be identified after surgical resection. To this end, noninvasive quantitative MRI has played an important role in TLE diagnosis and treatment of epilepsy since the early 1990s [5], particularly when surgery is being considered [6].

The typical MRI features of HS include hippocampal volume loss on T1-weighted (T1W) imaging and increased signal intensity on T2-weighted (or FLAIR) imaging [6, 7]. As hippocampal atrophy can also be an age-related change and increased T2 signal also presents with lesions other than HS [8], these two features achieved better detection of HS when used in combination [9–11]. However, unlike neuropathological examinations that report HS pattern or severity (e.g. ILAE type [4] or Wyler grading score [1]), MRI-based HS evaluation was generally binary (presence of HS or not). This may result from the lack of visual rating-based grading scale on MRI, which can serve as the target of model fitting with quantitative MRI features (as predictors). Although high resolution MRI [12–14] (e.g. 7 T MRI) helps to visualize the features of hippocampal substructures that were associated with pathological grading scales, it cannot be applied to practice due to its limited access in clinical routine.

In this regard, we proposed a study to quantify HS severity based on MRI features with two steps. First, a four-class grading scale of HS based on MRI was constructed, where two experienced neurosurgeons (with over 20 years of epileptic surgery) and one experienced neurologist at the Epileptic Center were involved for the visual ratings. Second, with the agreed HS rating on a TLE cohort, we fitted an ordinal regression model to map the association between the MRI features (hippocampal atrophy and increased FLAIR signal) and the HS grading score, resulting in a continuous index of HS. As the reliability of hippocampal segmentation tool also matters in detection of HS [15], we applied the recently validated software AccuBrain [16] for the quantification of hippocampal volume and FLAIR signal. The generated index of HS severity was compared with the visual rating categories of the raters for validation.

## Methods

### Subjects

We recruited 42 patients (21 male, 12–52 years, mean ± SD age 29 ± 10 years) with TLE that was refractory to medical therapy as diagnosed at the Epilepsy Center of Peking Union Medical College Hospital. The data collection was conducted retrospectively. The inclusion criteria were detailed as follows: (1) the semiology of the patients matched typical clinical manifestation of temporal lobe epilepsy; (2) the patients had undergone brain MRI (both 3D T1-weighted (T1W) scan and 2D FLAIR scan) before surgery; (3) the patients had evidence of ictal or interictal epileptiform activity arising from temporal lobe monitored with video electroencephalography (VEEG); (4) the patients had pre-surgery PET/CT examination that indicated hypometabolism in temporal lobe. (5) Indications of surgery for TLE were made through multi-department consultations for the patients. The exclusion criteria were: (1) patients with extra-temporal lobe epilepsy; (2) presence of other lesions on MRI, such as cavernous hemangioma and ganglioglioma; (3) the patients who only had 3D T1W scan or 2D FLAIR scan.

### Data acquisition

A 3 T MR imaging system (Discovery MR 750 scanner, GE Medical Systems) was used for image acquisition. Three-dimensional sagittal T1W images were acquired using a gradient echo sequence (BRAVO) with the following parameters: repetition time (TR) = 7.404 ms, echo time (TE) = 2.82 ms, flip angle (FA) = 12°, inversion time (TI) = 400 ms, matrix = 512 × 512, FOV = 512 × 512 mm^2^, number of slices = 180, slice thickness = 1.0 mm, no gap, spatial resolution = 0.4688 × 0.4688 × 1 mm^3^. Fluid-attenuated inversion recovery (FLAIR) sequences were acquired at the coronal plane with TR = 12,037.5 ms, TE = 123 ms, FA = 111°, TI = 2200 ms, matrix = 512 × 512, number of slices = 38, and slice thickness = 4.0 mm, spatial resolution = 0.4297 × 0.4297 × 4 mm^3^.

### Visual rating

Visual rating was performed by two neurosurgeons (W.D. and C.S.) with over 20 years’ experience in TLE surgery and an experienced neurologist (Q.L.) at the Epilepsy Center. A four-class grading system (0, normal; 1, mild; 2, moderate; 3 severe) was applied according to the raters’ experiences in surgery and each rater rated all the TLE patients independently. For each rater, the patients were rated twice at different time points, and a final judgement was confirmed based on these two ratings.

Classic signs of MRI-based HS visual rating include reduction of hippocampal volume on T1W images and increased signal observed on FLAIR images, which are generally examined on coronal slices perpendicular to the long axis of the hippocampus [6, 17]. The severity of HS (grading score) was further estimated as follows: (1) On T1W image, the patient will be rated as having severe HS if the hippocampal volume decreases by over 50%, and rated as moderate or mild HS for a reduction of hippocampal volume by 25% ~ 50% or < 25% respectively; if no obvious hippocampal volume reduction is found, the subject will be rated as normal as far as T1W is concerned; (2) On FLAIR image, the intensity within hippocampus was compared with the surrounding grey matter to rate the strength of increased signal as part of HS severity from the perspective of FLAIR-based rating; (3) the ratings of T1W and FLAIR images were finally integrated with a linear combination (the empirically defined weights: 0.8 for T1W-based rating and 0.2 for FLAIR-based rating) to generate a synthetic four-class grading score.

Images were also carefully examined by the investigators in light of the clinical and EEG data of each patient [10]. When the ratings from the three raters were ready, a simple voting was performed to make an agreed HS grading for each individual. To evaluate the inter-rater reliability of the three raters, the intraclass correlation coefficient (ICC) was also tested using Cronbach’s alpha [18].

### Image processing

Hippocampal volumetric analysis were performed with AccuBrain® (on T1W MRI scans), which has achieved the best performance in hippocampal segmentation among the existing automatic brain segmentation tools in a recent validation study [16]. The absolute volume of hippocampus was normalized by intracranial volume (ICV), and the resulting hippocampal volume ratio (% of ICV) was used as one predictor for model construction in the following section. Grey matter (GM) tissue of the whole brain was also segmented on T1W with AccuBrain. The masks of hippocampus (both left and right) and GM from segmentation were projected from the T1W image to the FLAIR image of the subject with affine registration [19]. The resulting masks were used to outline the hippocampal and GM regions, and thus to calculate the average intensity of hippocampus and GM on FLAIR image in a case-by-case manner. Here, the mean intensity of hippocampus (or GM) on FLAIR image was calculated by averaging the intensities of all the voxels within the hippocampus (or GM) mask of the specific subject. The relative intensity of hippocampus on FLAIR (calculated as the ratio of mean intensity of hippocampus to the mean intensity of GM within a specific subject, based on the hypothesis that the signals within the hippocampus mask and GM mask follow the Gaussian distribution with a single peak) was used as another predictor for model construction.

### Statistical analysis

The T1W-based predictor (hippocampal volume) and the FLAIR-based predictor (relative intensity of hippocampus) were first used to fit a model with the grading scale from visual rating as the outcome. Here, we flipped the right hippocampus to the left to enlarge the data size for model fitting (i.e. 84 hippocampal data in 42 TLE patients). As the grading scale is an ordinal variable, we applied ordinal regression (OR) to for model fitting. Among the OR approaches in the literature [20], we selected the threshold models (where an unobserved continuous variable is assumed to underlie the ordinal response) for examination, including Proportional Odds Model [21] (POM, a linear model extended from logistic regression), Neural Network based POM [21] (NNPOM, nonlinear generalization of POM), Support Vector Ordinal Regression with Explicit Constraints [22] (SVOREX), SVOR with Implicit Constraints [22] (SVORIM), Kernel Discriminant Learning for Ordinal Regression [23] (KDLOR), and Reduction from Ordinal Regression to Binary Support Vector Machine [24] (REDSVM). The implementation of these methods is available for free from the website of the authors of the OR review paper [20] (http://www.uco.es/grupos/ayrna/orreview).

A 5-fold cross-validation (where each fold was once used for testing and the remaining folds were used for training) was performed when fitting each of the five models. The model performance was comprehensively evaluated with three metrics: (1) mean accuracy (ACC), a rigorous metric that indicates the ratio of correctly predicted cases (of any class) among all cases; (2) mean absolute error (MAE) [25], the average deviation between predicted and actual targets in number of categories; (3) Spearman’s rank correlation (R), nonparametric correlation between the predicted and actual categories. The best model is expected to have the optimal balanced prediction performance (ACC + R - MAE) after parameter optimization. Also, the optimal model should also have as few parameters to train as possible to control overfitting [26] given the small sample size in this study.

To further generalize the ordinal outcome of the optimal model to a continuous HS index (HSI), we applied a weighted linear combination of the probability of each category (as generated by the optimal OR model):

1 $$ \mathrm{HSI}=\left({P}_1+2\ast {P}_2+3\ast {P}_3\right)/3 $$

Where *P*_1_, *P*_2_ and *P*_3_ indicate the probability of mild, moderate and severe HS for a specific subject; the probability of no HS (normal, *P*_0_) is not included for HSI calculation as it would have a term of (0*P_0_) that has no contribution to the formula. The HSI ranges from 0 to 1, where a larger HSI indicates more severe HS.

To evaluate the performance of HSI in differentiating visual rating-based HS categories, we compared the HSI of different visual rating-based categories with Mann-Whitney U test (especially between normal vs. mild, mild vs. moderate and moderate vs. severe HS). Also, Spearman’s rank correlation tests were performed to evaluate the consistency of HSI with the rating scores of the three raters.

## Results

According to the voted visual rating, there were 4 subjects with no HS (age 30.5 ± 11.0 years), 11 subjects with bilateral HS (age 26.6 ± 11.6 years), 15 subjects with left HS (age 27.3 ± 9.0 years), and 11 subjects with right HS (age 33.6 ± 8.2 years) in our TLE cohort. By flipping the hippocampal data of the study cohort, each hippocampus was rated with the four-class HS grading scale independently, with the group size and the relevant characteristics (e.g. age or onset years of TLE corresponding to a specific hippocampi) shown in Table 1. Regarding the voting of the visual ratings, at least two raters had agreement on HS grading for all the hippocampus, and the raters had better agreement on normal hippocampi (21 of 35 normal hippocampi agreed by all the three raters) than the other categories (e.g. 3 of 16 mild HS agreed by all the three raters). As shown in Table 2, the three raters achieved comparable intra-rater reproducibility (mean ICC of single measures 0.832) and the inter-rater reliability was relatively high (0.806 for single measures of ICC).

**Table 1:** Characteristics of the TLE patients in different HS categories

	Normal	Mild HS	Moderate HS	Severe HS
Group size by voting	35	19	22	8
Agreed by 2 raters	14	16	18	7
Agreed by 3 raters	21	3	4	1
Age, years, mean ± SD	30.1 ± 9.2	29.4 ± 11.0	27.9 ± 9.2	27.8 ± 12.2
Gender, male	18	7	13	4
Onset, years, mean ± SD	13.3 ± 8.2	11.4 ± 7.4	14.1 ± 9.8	15.1 ± 7.5

The displayed characteristics correspond to the flipped hippocampal data. HS, hippocampal sclerosis

**Table 2:** Intra-rater reproducibility and inter-rater reliability of the three raters

	ICC single measures (95% CI)	ICC average measures (95% CI)
Intra-rater reproducibility		
Rater W.D.	0.874 (0.825–0.912)	0.954 (0.934–0.969)
Rater Q.L.	0.878 (0.831–0.915)	0.956 (0.937–0.930)
Rater C.S.	0.744 (0.657–0.816)	0.897 (0.852–0.930)
Inter-rater reliability	0.806 (0.736–0.863)	0.926 (0.893–0.950)

The displayed ICC coefficients were all significant at the level of *p* < 0.001. *ICC* Intraclass correlation coefficient, *CI* Confidence interval

### Model selection with cross-validations

In general, the performances of the various ordinal regression models were very similar (Table 3). Although the KDLOR model achieved the best performance (in terms of ACC + R - MAE), it requires three parameters for model training. In this regard, we selected the traditional POM, which achieved similar performance with KDLOR and does not need any model parameters to train.

**Table 3:** Performance of different ordinal regression models in 5-fold cross-validations

Model	ACC	MAE	R	ACC + R - MAE	Optimized parameter(s)
POM	0.6919	0.3199	0.8428	1.2148	–
NNPOM	0.6801	0.3551	0.8391	1.1641	H = 5
SVOREX	0.6794	0.3441	0.8472	1.1825	k = 10, c = 10
SVORIM	0.6912	0.3324	0.8310	1.1898	k = 10, c = 10
KDLOR	0.7037	0.3081	0.8527	1.2483	k = 1, c = 0.1, u = 10^−6^
REDSVM	0.7037	0.3199	0.8386	1.2224	k = 10, c = 10

The mean ACC, MAE and R during the 5-fold cross-validations were displayed for each optimized ordinal regression (OR) model. The searching ranges of the model parameters follow the OR review paper [20]: H∈{5,10,20,30,40}, k∈{10^−3^,10^−2^,…,10^3^}, c∈{10^− 3^,10^− 2^,…,10^3^}, u∈{10^− 6^,10^− 5^,…,10^− 2^}. ACC, accuracy; MAE, mean absolute error; R, Spearman’s rank correlation; POM, proportional odds model; NNPOM, Neural network based on POM; SVOREX, support vector ordinal regression with explicit constraints; SVORIM, support vector ordinal regression with implicit constraints; KDLOR, kernel discriminant learning for ordinal regression; REDSVM, reduction from ordinal regression to binary support vector machine. H, the number of hidden neurons; k, the width of Gaussian kernel function; c, cost parameter of all SVM methods; u, additional parameter of KDLOR that is intended to avoid singularities in the covariance matrices [20].

### Comparison of HSI with visual rating

**Fig. 1:**
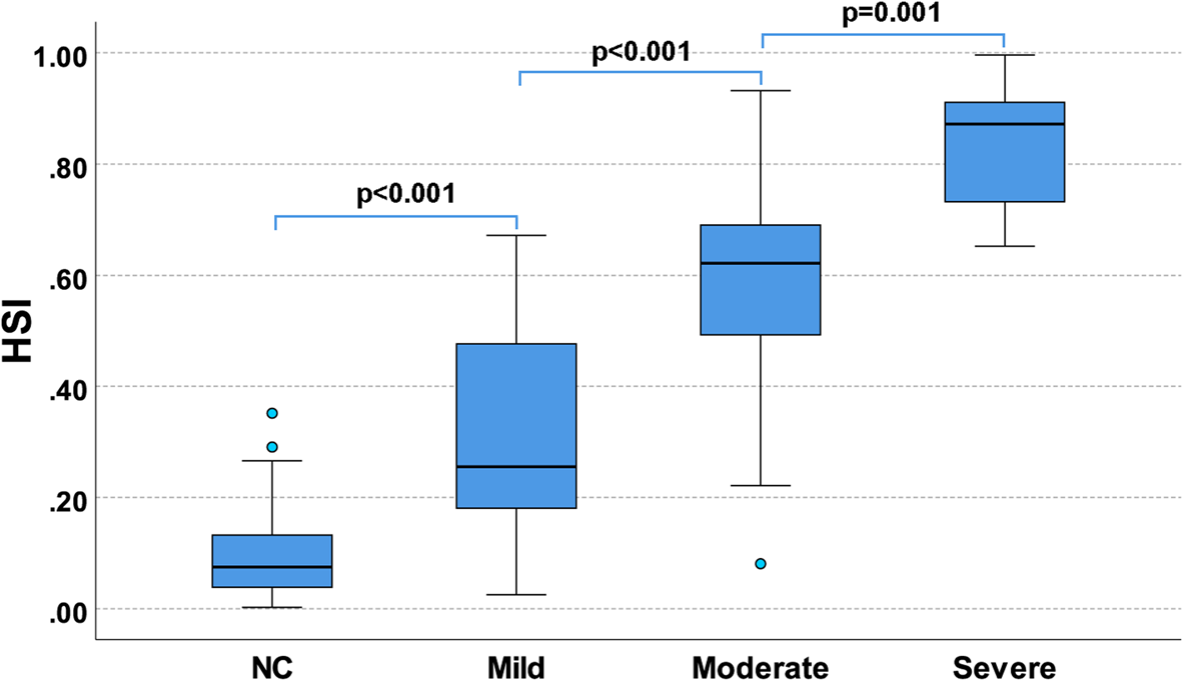
Boxplots of HSI for different visual rating-based categories

As POM was selected based on the performance of 5-fold cross-validations, we fitted a POM model based on the entire database and used the generated probability of each category to calculate the HSI according to Eq. (1). As shown in Fig. 1, the fitted HSI well differentiated the four categories, with little overlap of boxplots between any of the adjacent two categories. As confirmed by the Mann-Whitney U tests, the HSI was significantly larger in a more severe HS category than its adjacent HS category (NC < mild HS, *p* < 0.001; mild HS < moderate HS, *p* < 0.001; moderate HS < severe HS, *p* = 0.001). The HSI also performed well in a more general differentiation task (i.e. NC vs. HS, where the subgroups with mild to severe HS were combined as a whole HS group), with almost non-overlapped boxplots of NC and HS as shown in Supplementary Figure S1. In addition, the fitted HSI had high correlations with the grading scales of each rater (mean 0.786), which were comparable to the inter-rater correlations of the grading scales (mean 0.806) as shown in Table 4.

**Table 4:** Correlation between HSI and the HS categories identified from different raters

Spearman’s rank correlation	HSI	Rater D.W.	Rater Q.L.	Rater C.S.
HSI	1.000	0.823	0.817	0.717
Rater D.W.	0.823	1.000	0.878	0.777
Rater Q.L.	0.817	0.878	1.000	0.762
Rater C.S.	0.717	0.777	0.762	1.000

The correlation coefficients shown above were all significant at the level of *p* < 0.001.

## Discussion

In this study, for the first time we constructed an automated MRI-based index (HSI) to depict the HS severity in medically intractable TLE patients. The HSI was generated by fitting an optimized OR model with the hippocampal volume and hippocampal FLAIR signal as the predictors and the proposed four-class grading scales from visual inspection as the reference outcome.

Hippocampal volume and T2 or FLAIR signal have been widely applied (either individually [27, 28] or in combination [7, 9–11]) to differentiate hippocampus with HS from that without HS in TLE patients, but no study has applied these MRI features to evaluate HS severity or category, which is associated with postoperative outcome in TLE patients [3]. This might result from the lack of golden standard for visual inspection of HS severity as a reference. On the one hand, routine brain MRI generally provides very limited information compared with the neuropathological scans where visual grading scales are available [1, 4]. Although high resolution MRI scans (e.g. 7 T MRI) have been reported to depict pathological information of HS [12–14], they can rarely be used in clinical practice due to their high cost and poor accessibility. On the other hand, a distinct standard set for qualitative description of HS severity has not been available yet even for the well-established HS grading scales from neuropathological interpretations, as these pathological grading scales only provide general descriptions of the location and degree of cell loss and gliosis in hippocampus. In this regard, we turned to integrate the experiences of visual inspection for HS (on 3 T MRI) from epileptologists for a four-class grading scale to rate HS severity. Although the proposed grading scale was relatively subjective (i.e. the extent of hippocampal atrophy and FLAIR hyperintensity in hippocampus was scored only based on the experiences of the raters), we found that at least two of the three raters agreed on the rating of all the hippocampi of the cohort with a high inter-rater reliability (ICC: 0.814 for single measures and 0.929 for average measures).

Based on the voted rating scale from the three raters, we tried a series of OR models [20] to map the raters’ subjective but consistent experiences of visual inspection for HS into a quantitative model, with hippocampal volume and FLAIR intensity of hippocampus (% of GM intensity) as the independent variables. A comprehensive model selection was made among almost all the currently available threshold models of OR [20], with 5-fold cross-validations to increase the generalizability of the results. Although KDLOR achieved the best performance, it also required the most parameters to train, which induced more chances of overfitting [26]. Finally, we selected the POM model which ranked 3rd in model performance and did not fall behind much compared with the top two models (KDLOR and REDSVM, Table 3), as it required no model parameters to train. In POM, the ordinal outcome is generated by weighting the probability of each category, and these probabilities provide more information than the ordinal outcome (i.e. the predicted class) itself. In this regard, we integrated these probabilities into a continuous HS score (i.e. HSI, as shown in Eq. 1), where a more severe HS category would contribute more to the HSI score assuming that the probability of each category was the same. The resulting HSI well differentiated the visual rating-based HS categories (Fig. 1, Table 4), which indicated that the automated MRI-based index indirectivity learned the experiences of epileptologists on HS rating.

There are several limitations to this study that should be considered. First, the sample size of the study cohort is relatively small, and an external validation dataset is not available. However, we applied cross-validations and comprehensive model selections with least parameters to train, aiming to make the results as generalizable as possible. Further validations should be made in a larger cohort to test the reliability of the proposed POM model to calculate HSI. Of note, age and gender were not included in the model construction for the HSI, as they were not correlated with the grading score in the study cohort. Future work might also include these covariates if they present correlation with the target HS severity when a larger sample for model construction is available. Second, the proposed grading scale of HS severity from visual inspection was relatively subjective. However, the involved three raters achieved good intra-rater reproducibility and inter-rater reliability (Table 2), which indicated that a consistent visual rating can be realized under this grading scheme among experienced neurosurgeons and neurologists at the Epilepsy Center. In addition, regarding the type of T2 signal (one of the MRI features to measure HS), we applied normalized FLAIR intensity instead of T2 relaxometry although the latter one was more widely used in the existing studies. It was because the T2 relaxometry sequence was not available in our study cohort. As a recent study reported that T2 relaxometry was more sensitive than normalized FLAIR intensity to detect HS (when hippocampal atrophy was not considered) [29], future work should aim to test if T2 relaxometry would also contribute to a better evaluation of HS severity when hippocampal atrophy is also considered in the model. Furthermore, a comprehensive pathological validation of the proposed HSI was not available in this study, as only a small subset of the participants had the surgical samples (Supplementary Table S1) that could be used for evaluation of HS pathology due to the retrospective design of this study. Although the absolute certainty for the presence of HS cannot be assured without postresection outcomes, the distribution of HSI presented very little overlap between visual rating-based NC and HS groups (Supplementary Figure S1), indicating the potential of HSI as a screening tool before possible surgery. The consistency of MRI-based HSI with the surgical samples still warrant further validations with a larger sample of postresection outcomes. Finally, post-surgery outcomes of the TLE cohort were not yet available, and our following validations will focus on the associations between HSI and post-surgery outcomes to evaluate the use of HSI in clinical practice.

## Conclusions

In summary, this study proposed an MRI-based index (HSI) based on automatic quantification of hippocampal atrophy and increased FLAIR intensity to measure the severity of HS. The proposed HSI showed high consistency with the visual rating scales from experienced epileptologists. The HSI may help to evaluate HS severity in clinical practice, which warrants further validations in a larger sample of TLE patients with accessible post-surgery outcomes.

## Supplementary information

**Additional file 1: Figure S1.** Boxplots of HSI for NC and HS groups as identified by visual rating. HS = hippocampal sclerosis, HSI = HS index, NC = normal control. **Table S1**. Pathological data for a subset of the study cohort after tissue resection surgery

## Data Availability

The datasets used and analyzed during the current study are available from the corresponding author on reasonable request.

## Abbreviations

ACC: Accuracy
FA: Flip angle
FLAIR: Fluid-attenuated inversion recovery
FOV: Field of view
GM: Grey matter
HS: Hippocampal sclerosis
HSI: Hippocampal sclerosis index
ICC: Intraclass correlation coefficient
ICV: Intracranial volume
ILAE: International League Against Epilepsy
KDLOR: Kernel discriminant learning for ordinal regression
MAE: Mean absolute error
NC: Normal control
NNPOM: Neural network based proportional odds model
OR: Ordinal regression
POM: Proportional odds model
REDSVM: Reduction from ordinal regression to binary support vector machine
SVOREX: Support vector ordinal regression with explicit constraints
SVORIM: Support vector ordinal regression with implicit constraints
T1W: T1-weighted
TE: Echo time
TI: Inversion time
TLE: Temporal lobe epilepsy
TR: Repetition time
