# Supplementary material for: A quantitative MRI index for assessing the severity of hippocampal sclerosis in temporal lobe epilepsy
Source: BMC Med Imaging. 2020 Apr 25;20:42. doi: 10.1186/s12880-020-00440-z (PMC7183666; doi:10.1186/s12880-020-00440-z)
Supplement: Supplementary file 1 — Additional file 1: Figure S1. Boxplots of HSI for NC and HS groups as identified by visual rating. HS = hippocampal sclerosis, HSI = HS index, NC = normal control. Table S1. Pathological data for a subset of the study cohort after tissue resection surgery [file 12880_2020_440_MOESM1_ESM.docx]

**Supplementary material**


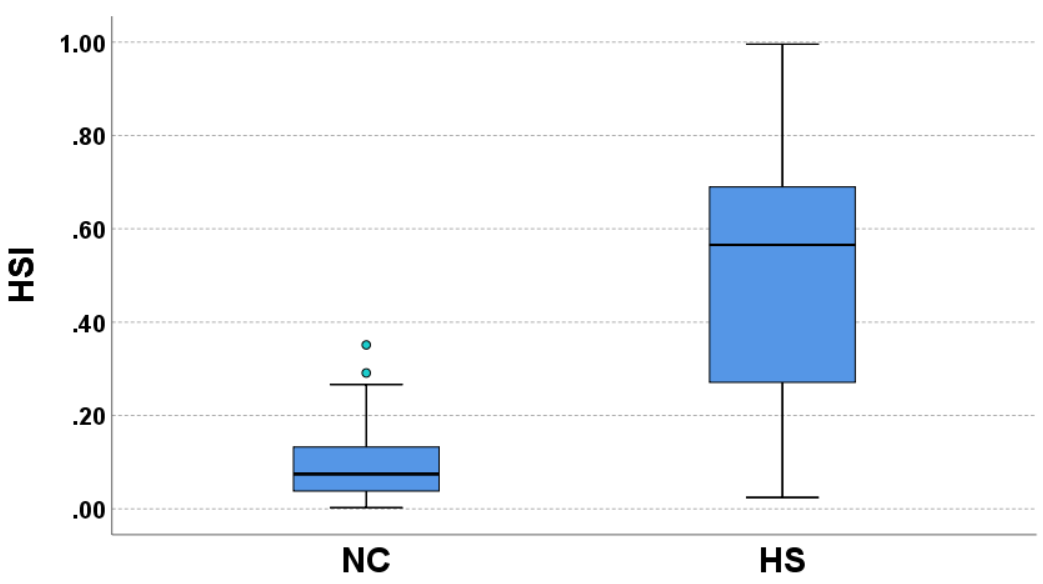


**Figure S1.** Boxplots of HSI for NC and HS groups as identified by visual rating. HS = hippocampal sclerosis, HSI = HS index, NC = normal control.

**Table S1**. Pathological data for a subset of the study cohort after tissue resection surgery

| Case ID | Surgery site | Pathology | Visual rating | HSI |
| --- | --- | --- | --- | --- |
| 1 | Right | Normal | Moderate | 0.3900 |
| 2 | Left | HS | Mild | 0.6612 |
| 3 | Left | HS | Moderate | 0.6899 |
| 4 | Left | HS | Mild | 0.3171 |
| 5 | Left | HS | Moderate | 0.5353 |
| 6 | Left | HS | Severe | 0.9200 |
| 7 | Left | HS | Moderate | 0.7522 |
| 8 | Left | HS | Moderate | 0.9326 |
| 9 | Left | HS | Mild | 0.6712 |
| 10 | Right | HS | Severe | 0.9958 |
| 11 | Right | HS | Moderate | 0.7146 |
| 12 | Right | HS | Moderate | 0.6138 |

Displayed are the cases where the hippocampal tissue was visible on the surgical samples. The range of HSI for a normal hippocampus is 0~0.2608, where the upper limit is defined with mean+1.96SD of HSI within the data that was voted as normal from the three raters. According to this normal range of HSI, the 11 cases with HS pathology were all identified correctly while the one case with normal pathology was mistaken as HS. HS = hippocampal sclerosis, HSI = HS index.
